# Child and Adolescent Mental Health Conditions and Crisis Management: Bespoke Education of Local Police Forces

**DOI:** 10.1192/bjo.2024.292

**Published:** 2024-08-01

**Authors:** Michael Foster, Haya Nadir

**Affiliations:** North Staffordshire Combined Healthcare NHS Trust, Stoke-On-Trent, United Kingdom

## Abstract

**Aims:**

The aim of this work was to improve police force understanding of the mental health difficulties of children and their management in challenging situations.

Nationally, approximately 18% of children aged 6–16 years are likely to have a mental disorder, with the frequency increasing in regions such as North Staffordshire where there are areas of significant deprivation. During the COVID pandemic, children's wellbeing, school attendance, and isolation all worsened resulting in a tripling of urgent referrals to some crisis mental health services. Owing to underfunding and reduced resources in the NHS, the police services have had to spend more time dealing with children's mental health crises, with some forces identifying insufficient training for understanding and de-escalating these emergencies.

It was hypothesized that the preparation and delivery of bespoke training sessions, coupled with pre- and post-assessments, would help enhance police understanding of the conditions and strategies in managing these crises.

**Methods:**

Extending previous approaches, each teaching session covered the presentation, diagnosis, and management of autism, conduct disorder, and emotional dysregulation in children, along with de-escalation. A pre- and post-session quiz was completed addressing each of the four topics. Data collection took place in October 2023 with 19 pairs of quizzes completed by local police and community support officers. Given the size and nature of the data, a non-parametric bootstrap resampling method was used to assess whether the teaching produced a statistically significant improvement in each topic and overall score.

**Results:**

Mean differences in scores with 95% confidence intervals (CIs) were obtained for the 19 pairs of quizzes for each mental health condition and all conditions taken together. With maximum of 16 marks possible in each condition, there were statistically significant improvements in mean scores for autism, 1.9, CI: [1.6,2.3]; conduct disorder, 2.2, CI: [1.7, 2.7]; emotional dysregulation, 2.4, CI: [2.0, 2.7]; and de-escalation, 0.9, CI: [0.5, 1.4], and for all conditions 7.4, CI: [6.5, 8.5].

**Conclusion:**

Bespoke children's mental health training sessions were found to deliver significant improvements in police knowledge of crisis management and de-escalation in children affected by autism, conduct disorder, and emotional dysregulation. Given the unprecedented demands on police services, training sessions of this kind could serve as a training tool to reduce both the intensity and duration of crises they have to handle. More sessions have already been requested within the authors’ local NHS Trust.

